# GENECODIS: a web-based tool for finding significant concurrent annotations in gene lists

**DOI:** 10.1186/gb-2007-8-1-r3

**Published:** 2007-01-04

**Authors:** Pedro Carmona-Saez, Monica Chagoyen, Francisco Tirado, Jose M Carazo, Alberto Pascual-Montano

**Affiliations:** 1 BioComputing Unit, National Center of Biotechnology (CNB-CSIC), C/Darwin 3, Campus Universidad Autónoma de Madrid, 28049 Madrid, Spain; 2 Computer Architecture Department, Facultad de Ciencias Físicas, Universidad Complutense de Madrid, C/Avenida Complutense S/N, 28040 Madrid, Spain

## Abstract

We present GENECODIS, a web-based tool that integrates different sources of information to search for annotations that frequently co-occur in a set of genes and rank them by statistical significance. The analysis of concurrent annotations provides significant information for the biologic interpretation of high-throughput experiments and may outperform the results of standard methods for the functional analysis of gene lists. GENECODIS is publicly available at .

## Abstract 2

GENECODIS, a web-based tool for finding annotations that frequently co-occur in a set of genes and ranking them by their statistical significance, is presented.

## Rationale

High-throughput experimental techniques such as DNA microarrays or proteomics are allowing researchers to study biologic systems from a global perspective. In many cases, the net result of these experiments is a large list of genes or proteins that are potentially interesting for the analyzed system, for example genes that are differentially expressed among normal and pathologic tissues. A logical further step in the analysis workflow is to translate such lists of significant genes into functional descriptors that help researchers in the process of elucidating the biologic meaning of their experimental results.

Since Khatri and coworkers introduced Onto-Express [1], several methods have been proposed within this context, aimed at interpreting and extracting biologic knowledge from large lists of genes or proteins. Most of these applications find biologic annotations that are significantly enriched in a list of genes with respect to a reference set, usually the whole genome or those genes used in a microarray. Using a specific source of information, for example Gene Ontology (GO) [2], those tools first find all of the GO terms associated with the set of analyzed genes. The number of appearances of each term is then determined in the input and reference lists, and a statistical test - usually the hypergeometric, χ^2^, bionomial, or Fisher's exact test - is used to compute *p *values, which are subsequently adjusted for multiple testing. The result of this analysis is a list of single biological annotations from a given ontology (for instance, GO terms) with their corresponding *p *values. Those terms with *p *values indicating statistical significance are representative of the analyzed list of genes and can provide information about the underlying biologic processes. Good reviews of such methods are available elsewhere [3,4].

Most of the currently available tools, however, are designed to evaluate single annotations, which means that they provide a list of annotations with their corresponding *p *values without taking into account the potential relationships among them. Finding relationships among annotations based on co-occurrence patterns can extend our understanding of the biologic events associated with a given experimental system. For example, a set of differentially expressed genes may be associated with the activation of biologic processes that are restricted to certain cellular organelles. Retrieving such associations provides meaningful and additional information for the interpretation of the experimental results.

In addition, the analysis of single annotations may show limitations in some cases. A simple motivating example of such limitations can be explained by using a hypothetical case of GO terms. There are categories such as 'signal transduction' that, although related to concrete aspects of the cell physiology, are associated with genes that are involved in disparate biologic processes, and therefore they may be annotated together with other terms such as 'cell proliferation' or 'apoptosis'. In this scenario, in a list of genes annotated as 'signal transduction' and 'cell proliferation', we may find that none of these terms are significant because a large number of genes in the genome belonging to each one of these categories are not included in the analyzed set. On the contrary, the co-occurrence of both categories might be significant if most of the genes simultaneously annotated with both terms are included in the list. This co-occurrence information reveals that a significant proportion of genes in the set are involved in specific signaling pathways related to cell proliferation. Therefore, relevant associations might be underestimated if only single annotations are taken into account.

These observations prompted us to develop GENECODIS, a web-based tool for finding sets of biological annotations that frequently appear together and are significant in a set of genes. It allows the integrated analysis of annotations from different sources (for example, KEGG pathways, Swiss-Prot keywords, GO, and InterPro motifs) and generates statistical rank scores for single annotations and their combinations. We believe that GENECODIS is an important extension of existing tools for the functional analysis of gene lists. GENECODIS is publicly available from the application website [5].

## The GENECODIS algorithm

**Figure 1:**
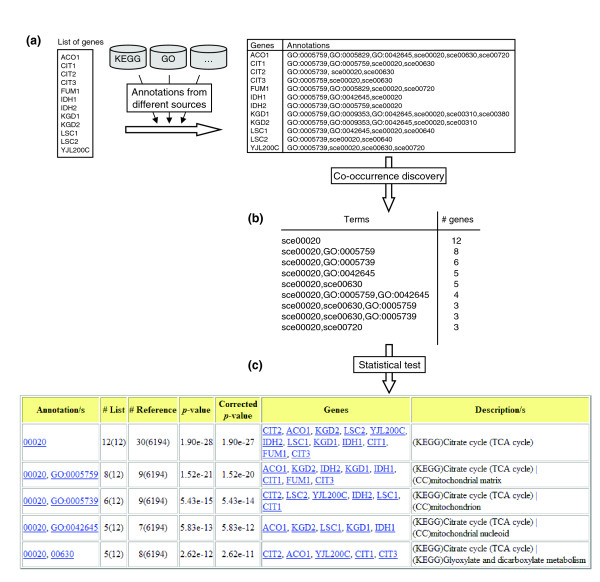
Overview of the methodology. **(a) **Annotations from several sources are assigned to genes in the input list. **(b) **The *apriori *algorithm is applied to find sets of annotations that frequently co-occur in the input list. **(c) **The statistical significance of each annotation or set of concurrent annotations is calculated based on its frequency in the input and reference sets. The figure illustrates an example in which a list of yeast genes is annotated with Gene Ontology (GO) terms for 'cellular component' and KEGG pathways. In the output table only the annotations that co-occur in more than five genes are shown.

The application that we propose is simple in its concept; it takes a list of genes as input and determines biological annotations or combinations of annotations that are over-represented with respect to a reference list. The novelty of this tool relies in the fact that, before computing the statistical test, it incorporates a new functionality to extract all combinations of annotations that appear in at least *x *genes, with *x *being a user-defined threshold (Figure 1 shows an overview of the methodology).

### Finding sets of terms that frequently appear together in a list of genes

To extract combinations of gene annotations, GENECODIS uses a modification to the methodology reported by Carmona-Saez and coworkers [6], which implements the *apriori *algorithm to extract associations among gene annotations and expression patterns.

The *apriori *algorithm was originally introduced by Agrawal and coworkers [7] and has been extensively used to extract association rules from transaction databases. This algorithm generates sets of elements that frequently co-occur in a database of transactions. Briefly, the procedure starts by determining the set of all single annotations ('itemset') that appear in at least *x *genes (also known as support threshold) from the list of interest and establish the frequent *k *itemsets, where *k *= 1. In the second iteration (*k *= 2), the set of frequent annotations found in the previous step is used to produce the new set of candidates of size 2 (2-itemset), and the database is scanned again to explore each gene and counting the frequency of each pair of annotations. However, if the set of annotations does not satisfy the minimum support constraint - that is, they do not occur in at least *x *genes - then they are not further considered to generate larger itemsets. The procedure continues until no more combinations are possible. At the end of this search all itemsets that contain the collection of annotations that co-occur in at least *x *genes are obtained (Additional data file 1).

In our previous work [6] we used the *apriori *algorithm to extract association rules among gene annotations and expression patterns. However, in this work we use it as the initial step in the methodology included in GENECODIS, namely the extraction of sets of annotation that frequently co-occur in a gene list.

It is important to note that increasing the number of different items (sources of annotation in this case) while decreasing the minimum support value can significantly multiply the number of concurrences and thus the computation time. Additional data file 1 contains a complete study of execution time and size of the itemsets for different support values in real datasets. Very extreme scenarios, such as extracting all possible combinations of terms that appear in at least one gene (support value of 1), is in many cases a computationally unfeasible task. For this reason we have provided the application with a minimum support value of 3, which is a reasonable threshold to extract significant biological information from gene lists.

### Statistical analysis

Once all combinations of annotations that appear in at least *x *genes have been extracted, the method counts the occurrence of each set of annotations in the list of genes and in a reference list. Note that for each set of concurrent annotations its frequency is calculated as the number of genes that are simultaneously co-annotated with those terms. By default, GENECODIS uses as a reference set all genes from the corresponding genome at the NCBI Entrez Gene database [8], but users can upload their own reference set (for example, genes in a chip). Then, a statistical test is applied to identify categories, and their combinations, that are significantly enriched in the list of genes. Two statistical tests are implemented in GENECODIS: the hypergeometric distribution and the χ^2 ^test of independence. For a detailed description of these methods in the context of the ontological analysis of gene lists, see the work of Draghici and coworkers [9] and the online help for the program.

The *p *values can then be adjusted for multiple tests using a simulation-based correction approach [10,11] or the false discovery method proposed by Benjamini and Hochberg [12]. For the simulation-based correction, a gene list of the same size of the input list is generated by randomly selecting genes from those used as reference. The frequent itemsets are then extracted (as described above) from this random list and their corresponding *p *values are calculated. This process is repeated 10,000 times and the corrected *p *value for each *k *itemset is calculated as the fraction of simulations having any *k *itemset with a *p *value as good as or better than the *p *value for that *k *itemset.

Therefore, the result of the analysis performed by GENECODIS consists of a list of annotations or combinations of annotations with their corresponding *p *values. Annotations exhibiting *p *values below a certain threshold can be considered significantly associated with the list of genes under study and can be used to discern the biologic mechanisms relevant to the experimental system.

## Implementation

GENECODIS is a web-based tool that is freely accessible from the application website [5]. It uses the Entrez Gene database [8] as the backbone data structure to link the functional annotations imported from GO together with the correspondences among gene identifiers (IDs). It allows users to upload gene lists using different IDs, including, for example, Gene Symbols, Entrez Gene, or Unigene IDs (more information about the identifiers supported for each organism can be found in the application website). If duplicated IDs are used in the input list, then they are treated as unique entries.

For each organism GENECODIS provides analysis of different annotations, including the three GO categories (biological process, cellular component, and molecular function), KEGG pathways, InterPro Motifs, and Swiss-Prot keywords. GO annotations for each gene are imported from the NCBI Entrez Gene database. GENECODIS allows users to select different levels of the GO hierarchy as well as GO Slim terms [13]. Information about metabolic pathways is imported from KEGG database [14], whereas Swiss-Prot keywords and InterPro motifs are imported from Swiss-Prot database.

Regarding the supported organisms, GENECODIS currently works with *Arabidopsis thaliana*, *Bos taurus*, *Caenorhabditis elegans*, *Danio rerio*, *Drosophila melanogaster*, *Gallus gallus*, *Homo sapiens*, *Mus musculus*, *Rattus norvegicus*, *Saccharomyces cerevisiae*, and *Schizosaccharomyces pombe*. More organisms and annotations will be systematically added in future versions of the application.

One relative limitation derived from the in-depth search performed is the increase in the computational cost and time as more annotation categories are analyzed. To tackle this limitation GENECODIS uses an efficient technique to extract frequent itemsets [6]. Additionally, GENECODIS runs on a 16-processor cluster, which guarantees the simultaneous use of the tool by multiple users.

## GENECODIS at work

We provide two examples showing the analysis performed by GENECODIS and how the results obtained as combinations of several biological annotations provide additional information that may be useful in the interpretation of high-throughput experimental data.

### Yeast data

To illustrate GENECODIS, we show the results obtained using data generated by Smith and coworkers [15]. They used oligonucleotide-based whole genome microarrays to measure gene expression levels in yeast during growth in oleate (peroxisome induction) and growth in glucose (peroxisome repression conditions). Using different clustering algorithms they identified 224 yeast genes whose expression patterns were similar to well known peroxisomal genes.

**Figure 2:**
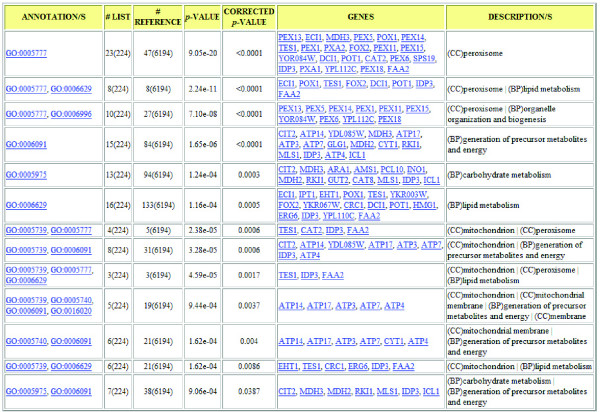
Screenshot depicting results of the analysis of yeast genes. The 'Annotation/s' column represents the Gene Ontology codes of annotations found in the list. The '# list' and '# reference' columns represent the number of genes in the input list and reference list for a given annotation, respectively. The 'Genes' column represents the set of genes in the input list showing a given annotation. The 'Description/s' column represents the textual description of annotations. CC refers to 'cellular component' and BP to 'biological process' categories. Only annotations with corrected *P *values ≤ 0.05 are shown. *P *values were calculated using the hypergeometric distribution and were corrected using the simulation-based approach.

The list of these 224 genes was re-analyzed using GENECODIS, selecting biological process (BP) and cellular component (CC) GO Slim annotations. The simultaneous analysis of both categories provided a global picture of the biological processes associated with the experimental system linked to cellular localization information (Figure 2). As was expected, the most significant category associated with this gene list was 'peroxisome' (CC). Other single categories that were highly representative were 'generation of precursor metabolites and energy' (BP), 'carbohydrate metabolism' (BP), and 'lipid metabolism' (BP), which is consistent with the observation that the shift to growth in the presence of oleate activates genes encoding enzymes that are involved in fatty acid degradation, allowing efficient use of the new carbon source [16].

In addition to these single-category significant annotations, GENECODIS revealed a new set of associations with a strong biologic meaning. For example, taking a closer look at the second and third categories with the lowest *p *values, we can see that a significant set of genes were co-annotated with 'peroxisome' (CC) and 'lipid metabolism' (BP), and 'peroxisome' (CC) and 'organelle organization and biosynthesis' (BP), respectively. These findings allow us to easily identify the set of peroxisomal genes that are specifically involved in each one of these two different biological processes. Among the genes co-annotated as 'peroxisome' (CC) and 'lipid metabolism' (BP) are the genes involved in the fatty acid β-oxidation pathway, such as *POX1*, *FAA2*, *ECI1*, *FOX2*, *POT1*, and *DCI1*. Among genes co-annotated as 'peroxisome' (CC) and 'organelle organization and biosynthesis' (BP) are the PEX genes, which are involved in peroxisome assembly [15] and are required for the increase in the number of these organelles during growth on oleate [16].

Another interesting set of annotations that show the usefulness of the application are those categories related to mitochondrial genes. Forty-eight out of 887 yeast genes annotated as 'mitochondrion' (CC) were present in the list, and therefore this annotation exhibited a *p *value of 0.0248 (simulation corrected *p *value = 0.2; Additional data file 2). Consequently, based on the statistical test, this annotation is not considered significant. Nevertheless, GENECODIS was able to identify a set of significant co-annotations related to mitochondrial genes. For example, 6 out of 21 yeast genes that were simultaneously annotated with 'mitochondrion' (CC) and 'lipid metabolism' (BP) were present in the list, and this co-annotation exhibited a *p *value of 0.000162 (simulation corrected *p *value = 0.0086). Among these genes was, for example, the *CRC1 *gene, which is a mitochondrial inner membrane carnitine transporter that is required for carnitine-dependent transport of acetyl-coenzyme A from peroxisomes to mitochondria. In the same way, the co-annotation of 'mitochondrial membrane' (CC) and 'generation of precursor metabolites and energy' (BP) related to a subset of genes that are component of the mitochondrial respiratory chain was found to be significant, with a simulation corrected *p *value of 0.004.

Although fatty acid β-oxidation in *Saccharomyces cerevisiae *is restricted to peroxisomes, the association of mitochondrion related categories to this set of genes is highly consistent with the important role of these organelles in the metabolism of β-oxidation products. Acetyl-coenzyme A, the final product of the fatty acid β-oxidation pathway in peroxisomes is transported to the mitochondria for the final oxidation to CO_2 _and H_2_O [17]. In this way, peroxisomal fatty acid β-oxidation demands a functional mitochondrial electron transport chain for energy production, and either functional peroxisomes and mitochondria are required for growth in the presence of oleate [15].

### Human data

To provide a second example of the functionality of GENECODIS, we analyzed a set of 85 human genes expressed in testis reported by Su and coworkers [18]. This dataset was also used by Zhang and colleagues [19] to illustrate the performance of the GOTree Machine (GOTM) software, and therefore it represents a good test case for our method. Zhang and colleagues, using GOTM, reported four main groups of GO biological process annotations related to the testis gene cluster: categories related to cell proliferation, cell cycle, mitosis, and meiosis; categories related to testis specific development; categories related to protein phosphorylation; and categories related to glycerolipid metabolism.

**Figure 3:**
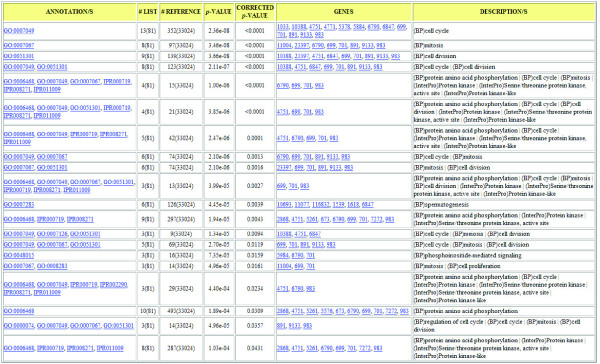
Screenshot depicting results of the analysis of human genes. GENECODIS results from the analysis of Gene Ontology CC ('cellular component') and InterPro motifs in the human gene set. Only annotations with corrected *P *values ≤ 0.05 are shown.

We used our tool to analyze this set of genes using the GO biological process categories and InterPro motifs that appear in at least three genes. The most significant concurrences are shown in Figure 3. Similar results to those reported by Zhang and coworkers [19] were obtained by GENECODIS, except for the case of categories related to glycerolipid metabolism, which were not extracted because they were present in only two genes. In addition, GENECODIS was able to provide new information for the functional interpretation of this set of genes. For example, the fifth association revealed that a significant set of genes in the analyzed list were co-annotated with 'protein amino acid phosphorylation' and 'cell cycle' GO biological process categories and contained protein kinase motifs. The importance of this observation is the explicit connection between 'protein amino acid phosphorylation' and 'cell cycle' categories.

In order to explain the 'protein phosphorylation' category in the context of the phenotypic feature of the gene cluster, Zhang and colleagues [19] remarked that, 'spermatozoa undergo a series of changes before and during egg binding to acquire the ability to fuse with the oocyte. These priming events are regulated by the activation of compartmentalized intracellular signaling pathways, which control the phosphorylation status of sperm proteins.'

Results provided by GENECODIS complement this finding and point out that, in this particular case, the 'protein phosphorylation' category is mainly related to proteins that are involved in cell cycle. Indeed, activation and inhibition of many key regulators of cell cycle are carried out by phosphorylation/dephosphorylation events.

This finding can be confirmed by examining the genes that were co-annotated with both categories: *CDC2 *(Entrez Gene ID: 983), aurora kinase A (Entrez Gene ID: 6790), *NEK2 *(Entrez Gene ID: 4751), *BUB1 *(Entrez Gene ID: 699), and *BUB1B *(Entrez Gene ID: 701). All of these have been associated with testis tissues and cell proliferation events in previous studies. For example, the *NEK2 *gene is predominantly expressed in spermatocytes and appears to be associated with meiotic chromosomes in these cells [20]; expression of the gene *BUB1B *in testis decreases with advancing age, and it may play a role in regulating infertility [21].

These two examples illustrate the type of information provided by GENECODIS, which can be useful in helping researchers to interpret large lists of genes generated by high-throughput experimental techniques.

## Discussion

High-throughput experimental techniques, such as DNA microarrays, have opened new ways to study biological systems from a global perspective. In many cases, these techniques generate huge amounts of data in the form of large gene or protein lists that share a common property, for example genes that are differentially expressed among pathologic and normal tissues. These data can provide a basis for the characterization of unknown genes, and at the same time they are also the basis for elucidating the biological processes associated with the experimental system. Methods based on the ontological analysis of such lists of genes have proved to be very useful tools for the analysis and interpretation of the underlying biological mechanisms.

However, most of the current applications for functional profiling essentially use the same general approach and generate statistical scores for single annotations. They mainly differ on aspects such as the statistical test used, supported annotations and organisms, the gene identifiers that they are able to manage, and visualization capabilities. Indeed, a relevant conclusion of a review of such tools recently reported by Khatri and Draghici [3] was that it would be more beneficial if future applications expand the current approach rather than providing endless variations of the same idea.

GENECODIS was designed to expand the biological enrichment of annotations by adding the possibility of extracting not only single enriched categories, but also significant combinations of them. To the best of our knowledge there is no other tool available in the field that integrates information from different sources in a flexible way for concurrent enrichment studies. A comparison of GENECODIS with related tools [1,22-25] and an example with test data [26] is provided in Additional data file 3. We hope that this tool will help by complementing available analysis tools for the large genome research community.

## Additional data files

The following additional data are available with the online version of this article. Additional data file 1 contains an illustrative example of the GENECODIS algorithm in operation. Additional data file 2 contains the results obtained by GENECODIS in the analysis of the yeast and human gene sets. Additional data file 3 provides a description of a comparative analysis of the results provided by GENECODIS and other related tools.

## Supplementary Material

Additional data file 1 A file containing an illustrative example of the GENECODIS algorithm in operation. Click here for file

Additional data file 2 A file containing the results obtained by GENECODIS in the analysis of the yeast and human gene sets. Click here for file

Additional data file 3 A compressed file containing a description of a comparative analysis of the results provided by GENECODIS and other related tools. Click here for file
