# Supplementary material for: GENECODIS: a web-based tool for finding significant concurrent annotations in gene lists
Source: Genome Biol. 2007 Jan 4;8(1):R3. doi: 10.1186/gb-2007-8-1-r3 (PMC1839127; doi:10.1186/gb-2007-8-1-r3)
Supplement: Additional data file 1 — A file containing an illustrative example of the GENECODIS algorithm in operation. [file gb-2007-8-1-r3-S1.pdf]

Finding sets of annotations that frequently co-occur in a list of genes

In this document we illustrate, with a straightforward example, the GENECODIS algorithm for finding sets of annotations that frequently co-occur in a gene list.

Given a list of genes, the first step is to retrieve annotations for each gene from the selected databases.

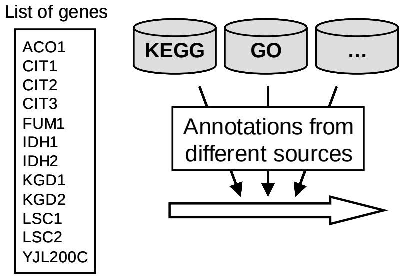

genes	Annotations
ACO1	GO:0005759,GO:0005829,GO:0042645,sce00020,sce00630,sce00720
CIT1	GO:0005739,GO:0005759,sce00020,sce00630
CIT2	GO:0005739, sce00020,sce00630
CIT3	GO:0005759,sce00020,sce00630
FUM1	GO:0005759,GO:0005829,sce00020,sce00720
IDH1	GO:0005759,GO:0042645,sce00020
IDH2	GO:0005739,GO:0005759,sce00020
KGD1	GO:0005759,GO:0009353,GO:0042645,sce00020,sce00310,sce00380
KGD2	GO:0005759,GO:0009353,GO:0042645,sce00020,sce00310
LSC1	GO:0005739,GO:0042645,sce00020,sce00640
LSC2	GO:0005739,sce00020,sce00640
YJL200C	GO:0005739,sce00020,sce00630,sce00720

In this example, KEGG and GO Cellular Component annotations have been associated to a set of yeast genes. This set of annotated genes can be treated as a transaction database, in which genes are transactions and annotations are itemsets using the classical nomenclature in the field of association rules. This transaction database can be mined to extract frequent sets of annotations that co-occur in at least *x* genes.

Given a minimum support value, let say *x*=3, the first step of the algorithm is to extract all annotations that occur in at least 3 genes (frequent 1-itemsests);

Annotations	# genes	genes
GO:0005759	8	ACO1,CIT1,CIT3,FUM1,IDH1,IDH2,KGD1,KGD2
GO:0042645	5	ACO1,IDH1,KGD1,KGD2,LSC1
sce00020	12	ACO1,CIT1,CIT2,CIT3,FUM1,IDH1,IDH2,KGD1,KGD2,LSC1,LSC2,YJL200C
sce00630	5	ACO1,CIT1,CIT2,CIT3,YJL200C
sce00720	3	ACO1,FUM1,YJL200C
GO:0005739	6	CIT1,CIT2,IDH2,LSC1,LSC2,YJL200C

Using these frequent 1-itemsets we generate the set of frequent 2-itemsets, that is, all pairs of annotations that co-occur in at least three genes;

Annotations	# genes	genes
GO:0005759,GO:0042645	4	ACO1,IDH1,KGD1,KGD2
GO:0005759,sce00020	8	ACO1,CIT1,CIT3,FUM1,IDH1,IDH2,KGD1,KGD2
GO:0005759,sce00630	3	ACO1,CIT1,CIT3
GO:0042645,sce00020	5	ACO1,IDH1,KGD1,KGD2,LSC1
sce00020,sce00630	5	ACO1,CIT1,CIT2,CIT3,YJL200C
sce00020,sce00720	3	ACO1,FUM1,YJL200C
sce00020,GO:0005739	6	CIT1,CIT2,IDH2,LSC1,LSC2,YJL200C
sce00630,GO:0005739	3	CIT1,CIT2,YJL200C

In the next step, we generate frequent 3-itemsets;

Annotations	# genes	genes
GO:0005759,GO:0042645,sce00020	4	ACO1,IDH1,KGD1,KGD2
GO:0005759,sce00020,sce00630	3	ACO1,CIT1,CIT3
sce00020,sce00630,GO:0005739	3	CIT1,CIT2,YJL200C

This procedure is continued until no more combinations are possible. In our example we have no 4-itemsets that occur in at least 3 genes so the process finish here.

From this set of frequent sets of annotations, those that contain redundant information are filtered. A frequent itemset can be defined as a redundant itemset if it is a subset of a larger itemset with equal or greater support value. That is, if a set of annotations, let say {A, B}, is a subset of longer set {A,B,C} and is associated to the same set or a subset of genes, the set of annotations {A, B} can be removed without loosing information.

After this process the frequency of each set of annotations in computed in the reference list and a *p*-value is calculated. In this way, the output of GENECODIS in this example will be the following one;

ANNOTATION/S	# LIST	# REFERENCE	*p*-VALUE	GENES	DESCRIPTION/S
00020	12(12)	30(6194)	1.90e-28	CIT2, ACO1, KGD2, LSC2, YJL200C, IDH2, LSC1, KGD1, IDH1, CIT1, FUM1, CIT3	(KEGG)Citrate cycle (TCA cycle)
00020, GO:0005759	8(12)	9(6194)	1.52e-21	ACO1, KGD2, IDH2, KGD1, IDH1, CIT1, FUM1, CIT3	(KEGG)Citrate cycle (TCA cycle) | (CC)mitochondrial matrix
00020, GO:0005739	6(12)	9(6194)	5.43e-15	CIT2, LSC2, YJL200C, IDH2, LSC1, CIT1	(KEGG)Citrate cycle (TCA cycle) | (CC)mitochondrion
00020, GO:0042645	5(12)	7(6194)	5.83e-13	ACO1, KGD2, LSC1, KGD1, IDH1	(KEGG)Citrate cycle (TCA cycle) | (CC)mitochondrial nucleoid
00020, 00630	5(12)	8(6194)	2.62e-12	CIT2, ACO1, YJL200C, CIT1, CIT3	(KEGG)Citrate cycle (TCA cycle) | (KEGG)Glyoxylate and dicarboxylate metabolism
00020, GO:0005759, GO:0042645	4(12)	4(6194)	4.04e-11	ACO1, KGD2, KGD1, IDH1	(KEGG)Citrate cycle (TCA cycle) | (CC)mitochondrial matrix | (CC)mitochondrial nucleoid
00020, 00630, GO:0005759	3(12)	4(6194)	5.55e-08	ACO1, CIT1, CIT3	(KEGG)Citrate cycle (TCA cycle) | (KEGG)Glyoxylate and dicarboxylate metabolism | (CC)mitochondrial matrix
00020, 00630, GO:0005739	3(12)	3(6194)	2.22e-08	CIT2, YJL200C, CIT1	(KEGG)Citrate cycle (TCA cycle) | (KEGG)Glyoxylate and dicarboxylate metabolism | (CC)mitochondrion
00020, 00720	3(12)	9(6194)	9.09e-07	ACO1, YJL200C, FUM1	(KEGG)Citrate cycle (TCA cycle) | (KEGG)Reductive carboxylate cycle (CO2 fixation)

Notes about execution time and number and size of itemsets

It is important to mention that although the application allows users to select different sources of gene annotations there are combinations of annotations that, although can be interesting to explore in some cases, may not be very meaningful from the biological point of view. For example KEGG pathways and GO Biological Process annotations are related to similar biological aspects of genes, and combinations among them rather than provide significant information can obscure the interpretation of the data by increasing the number of associations. On the contrary, others combinations such as co-occurrence patterns among biological processes and cellular components, or molecular functions

and sequence motifs can be very relevant to get a richer picture of the biology of the system. Here, the user has to choose those annotations that are more interesting to explore in a given context, knowing that the execution time and the number of annotations may increase with the number of different annotations selected in the same analysis.

To know the maximum number of combinations, that is, the longest *k*-itemset that can be expected in a set of differentially expressed genes we have to look for the gene that contains the largest number of annotations in the set. If that particular gene contains, for example 20 annotations, then 20 is the maximum value of *k* that we would obtain in the dataset. If this set of *k* annotations is simultaneously present in only one gene, then to obtain a *k*-itemset we need a support value of one. If the set of *k* annotations is present in two genes it would be obtained with support value of two, and so on. In this way we can say that the maximum value of *k* will depend on the number of different annotations simultaneously selected in one analysis and the support value used.

In GENECODIS the *k*-itemset with the highest value of *k* corresponds to the largest set of annotations that are simultaneously present in at least three genes. This restriction is imposed in order to select significant associations in a reasonable amount of time without having a combinatorial explosion in the search space. In addition, requesting associations in at least three genes seems to makes sense in order to derive biological meanings.

Below there are some graphs about the relationship between the number of combinations (values of *k*) and minimum support for the yeast and human data set discussed in the manuscript:

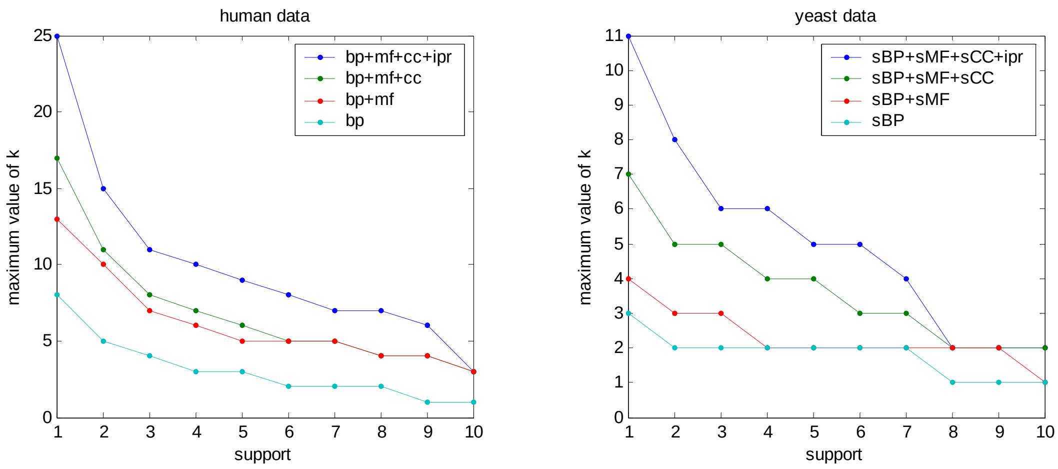

The graph on the left contains the data from the analysis of human data and the graph on the right shows results from the analysis of the yeast data set. The Y-axis represents the length of the maximum set of annotations (maximum value of *k*) found for each value of support (X-axis). For each dataset, we performed four different experiments using increasing sources of annotations. The legend of each figure represents each experiment indicating the annotations that were simultaneously analyzed (bp: GO biological process, cc: GO cellular component, mf: GO molecular function, ipr: interPro motifs, sBP: GO Slim biological process, sCC: GO Slim cellular component, sMF: GO Slim

molecular function). As expected, the maximum value of *k* increases when support value is decreased.

The execution times for extracting combinations of annotations may vary depending on the number of selected categories and the support values. Increasing the support value decreases the number of sets and, therefore, the execution time. As a matter of fact, that is the essence of the *apriori* algorithm. Below there are some plots about execution times and number of itemsets generated from the analysis of the human and yeast dataset:

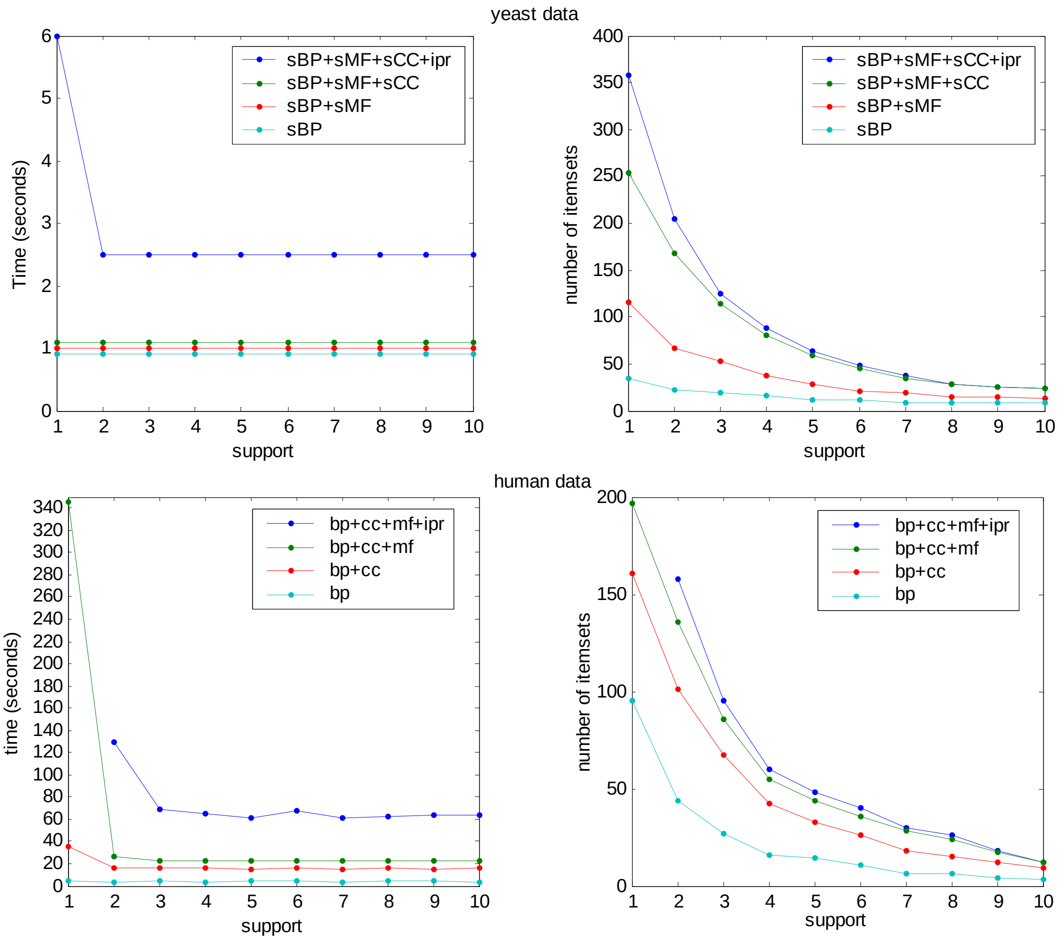

The figures reflect the execution times and number of itemsets versus support value. Figures on the top were generated from the analysis of the yeast dataset while the two plots on the bottom show the results from the analysis of the human data.
Times are calculated only for the *apriori* algorithm. Some additional time is also used in submitting the job to one of the computing clusters, processing the files and calculating statistics, although it was not included in the plots since they represent a minimum, almost constant fraction of the whole process.
An interesting point to mention is that in the case of Human data set, it was not possible to include information for support value of one due to the high number of combinations that were generated. This is a clear example where the *apriori* algorithm is useful since it allows a drastic reduction of the search space by taking into account those combinations of annotations that are frequently present in the dataset.

It is also important to note that although in many applications the extraction of frequent itemsets can be a very computational expensive methodology, the case of biological annotations is not one of the most limiting applications. This is probably due to the fact that genes tend to have a sparse and modular functional organization.

Some works have study the theoretical complexity of the apriori algorithm. GENECODIS only uses the frequent itemset search stage of the whole Apriori algorithm. The problem for an average case can be formulated as follow:

Considering a transaction that has *I* items. During the kth pass of the algorithm, this transaction has $C = \binom{I}{k}$ potential candidates that need to be checked against the candidate hash tree (which is the most used data structure to accelerate the search for potential candidates). The average number of leaf nodes in the hash tree is L = M/S. Where M is the total number of candidate items (annotations) and S is the average number of candidates at the leaf node of the hash tree. Taking into account that C is the average number of candidates at each transaction (genes in the list), the number of distinct leaf visited per transaction is $V_{C,L}$, and the computation time per transaction for visiting the hash tree is:

$T_{trans} = C \text{ x } T_{traversal} + V_{C,L} \text{ x } T_{check}$

Where $T_{traversal}$ is the cost of hash tree traversal per potential candidate, T_{check} is the cost for checking at the leaf with S candidates and $V_{C,L}$ is the expected number of leaves visited with C potential candidates and L leaves.
So, the run time of the algorithm for processing N transactions (genes) is:
$T = N\text{x } T_{trans} + O(M)$
$T = N \text{ x } C \text{ x } T_{traversal} + N \text{ x } V_{C,L} \text{ x } T_{check} + O(M)$
Where O(M) is the complexity for creating the hash tree.
