# Supplementary material for: GENECODIS: a web-based tool for finding significant concurrent annotations in gene lists
Source: Genome Biol. 2007 Jan 4;8(1):R3. doi: 10.1186/gb-2007-8-1-r3 (PMC1839127; doi:10.1186/gb-2007-8-1-r3)
Supplement: Additional data file 3 — A compressed file containing a description of a comparative analysis of the results provided by GENECODIS and other related tools. [file gb-2007-8-1-r3-S3.zip › Additional_file3/additionalFile3.pdf]

Comparison of GENECODIS with related tools

In the last few years several functional profiling tools have been developed in order to assist in the interpretation of data derived from high-throughput experiments (for a review see [3]). The main contribution of GENECODIS with respect to other related tools is that it is able to search and extract enriched co-occurrences of annotations in a unified analysis. To the best of our knowledge there is no other tool available in the field that integrates information from different sources in a flexible way for concurrent enrichment studies.

In addition to the lack of concurrent analysis, most of the available methods are exclusively orientated to use annotations from Gene Ontology and they manage each annotation independently from other categories of the ontology. The integration of various types of data from various sources is potentially more useful than the use of any single database and can offer a more complete picture of the biology underlying the experimental system [3]. In spite of this, only a few of the available tools are able to integrate information from diverse biological databases. Among those able to use annotations from several sources are Onto-Express [1], FatiGO plus in the Babelomics software [22], GeneMerge [23], DAVID [24] and WebGestalt [25]. Table 1 shows a general comparison among these methods in terms of the statistical test they use, organisms, different annotations they support and the potential to establish cross-relationships among different annotations.

Table 1. Scope column refers to the number of different annotations that can be analyzed simultaneously. The rest of columns are self-explanatory. *In GeneMerge only annotations that are used for at least two organisms are shown in the "annotations supported" column. ** WebGestalt allows multiple categories only for GO annotations. MTP: Multiple testing correction method.

Tool	Statistical Model (MTP)	Annotations supported	Organisms	Scope	Term co-occurrences
FatiGO+ (Al-Shahrour *et al*., 2005)	Fisher's exact test (Step-down minP, FDR)	Gene Ontology, KEGG pathways, Interpro Motifs, SwisProt keywords, Transcription factors, cis-regulatory elements	*A. thaliana*<br>*C. elegans*<br>*D. melanogaster*<br>*G. gallus*<br>*H. sapiens*<br>*M. musculus*<br>*R. norvegicus*<br>*S. cerevisiae*<br>*S. coelicolor*	Multiple categories	No
Onto-Express (Khatri *et al*., 2002)	Hypergeometric, Binomial, Fisher's exact test, χ^2 (Sidák, Holm, Bonferroni, FDR)	Gene Ontology, KEGG pathways, chromosome regions	more than 20 organisms	Multiple categories	No
GeneMerge* (Castillo-Davis *et al*., 2003)	Hypergometric (Bonferroni)	Gene Ontology, KEGG pathways, Chromosomal Location	20 different organisms	One category	No
DAVID 2006 (Dennis *et al*., 2003)	Fisher's exact test (None)	Gene Ontology, Protein Domains, Pathways, General Annotations, Functional Categories, Functional Interaction, Literature Diseases	more than 20 organisms	Multiple categories	No
WebGestalt** (Zhang *et al*., 2005)	Hypergometric, Fisher's exact test (None)	Gene Ontology, KEGG pathways, BioCarta pathways, Protein Domains	*H. sapiens*<br>*M. musculus*	Multiple Categories	No
GENECODIS	Hypergometric, χ^2 (simulation, FDR)	Gene Ontology, KEGG pathways, Interpro Motifs, SwisProt keywords	*A. thaliana*<br>*B. taurus*<br>*C. elegans*<br>*D. melanogaster*<br>*D. rerio*<br>*G. gallus*<br>*H. sapiens*<br>*M. musculus*<br>*R. norvegicus*<br>*S. cerevisiae*<br>*S. Pombe*	Multiple categories	Yes

In this context, we have only identified a related application that provides inter-relationships among gene annotations from different sources: the functional annotation clustering module included in the new version of the DAVID software package (DAVID 2006: http://david.abcc.ncifcrf.gov/). This piece of software uses kappa statistics to create gene-to-term similarity matrices based on the genes that are annotated with each term. This type of matrix is then explored by fuzzy heuristic clustering

algorithms in order to find groups of terms that share common genes. Nevertheless, this tool is mainly an exploratory method and different results are obtained when using different parameters. Therefore, even if this module may provide interesting results this methodology is still far from being a biological enrichment tool. GENECODIS, on the other hand, is designed to obtain the main significant sets of annotations that frequently co-occur in a set of genes and assign them a significance rank score.

A direct face to face comparison of GENECODIS with other related tools is far from being trivial because the results they provide are different in nature: the statistical test they use, multiple-test correction, level of the ontology or selection of several free parameters [26]. Nevertheless, in order to provide a qualitative comparison we have developed two different levels of analysis: one using a fictitious test-organism with characteristics compatible with several real organisms and the other one by a direct comparison with a real data set.

In the case of the analysis with the fictitious organism, we adopted the idea introduced by Vencio *et al*. [26] and tried the analysis using GeneMerge [23], which is a good representative of biological enrichment tools and, in addition, it allows users to use arbitrary ontologies.

Let's assume that this test-organism has 4000 genes which are annotated with terms from a synthetic ontology. This ontology has two different categories: the first category is related to biological processes and the second category is related to cellular components. Let's now focus on an arbitrary annotation in the first category, GO1:BP, that is associated to 400 genes and GO1:X define other terms different from GO1:BP. The second category, GO2:CC, is associated to 150 genes, and GO2:X describes all genes that have other terms different from GO2:CC. In this hypothetical genome there are ten genes that codify proteins that are localized in the cellular compartment

described by GO2:CC, and, at the same time, they are involved in the biological process described by GO1:BP, therefore these genes are simultaneously co-annotated with GO2:CC and GO1:BP. Suppose that a microarray experiment is carried out and 200 genes show a significant expression. Let's assume that this experiment activates the expression of genes involved in the biological process GO1:BP but whose proteins are exclusively localized in the cellular component GO2:CC. Therefore, the set of 200 genes includes the ten genes co-annotated with GO1:BP and GO2:CC and 190 genes annotated with other terms (GO1:X and GO2:X). GeneMerge, which uses the hypergeometric distribution, yields a *p*-value=0.99 for category GO1:BP and a *p*-value=0.21 for GO2:CC. Therefore, none of these terms are found as significantly enriched in the list of genes. Nevertheless, GENECODIS gives a *p*-value= 7.85E-14 for the co-annotation of GO1:BP and GO2:CC, indicating that, although each independent annotation is not significant, the list of genes is significantly enriched in genes simultaneously co-annotated with both categories.

With standard methods we probably do not get a connection of these two annotations with the experimental system, which can be relevant information for the interpretation of the experimental results. Indeed, all genes from this artificial genome that are localized in the cellular compartment GO2:CC and are involved in the biological process GO1:BP are found in the list of significant genes. This simple example shows the additional information that GENECODIS might provide with respect to standard methods. All information about the data and the results with both methods are included in the TestData excel file.

We also repeated the analysis of the yeast data with GeneMerge [23] and DAVID [24] software packages since they are well established applications for ontological

analysis of gene lists. Both tools provide similar sets of results consisting of a list of single annotations with their corresponding *p*-value.

In a first step we used DAVID module for biological enrichment to perform an analysis using GO Biological Process and GO Cellular Component that appear in at least three genes. All significant single annotations found by GENECODIS were also generated by the DAVID software, and categories such as "mitochondrion", "peroxisome", "peroxisome organization and biogenesis" or "cellular lipid metabolism" appear as significant. Nevertheless, from such a list of annotations one can not directly derive information about potential connections among different categories, such as the associations among "cellular lipid metabolism" and "peroxisomes" or the mitochondrial related categories commented above. Only a careful search and enumeration of genes that are annotated with those terms could provide clues about these associations. Because DAVID does not apply multiple testing corrections, we decided to use GeneMerge to complement these results. A significant difference was obtained with "mitochondrion" category which was not significantly reported after multiple testing correction (bonferroni corrected *p*-value = 1). This is in agreement with the results obtained by GENECODIS, but in this case, we can not extract any information about potential connections between "mitochondrion" and other categories (see Yeast_DAVIDResults and Yeast_GeneMergeResults excel files for full results).

As a final comparison, we also used DAVID package for functional annotation clustering in order to establish groups of related annotations based on similar sets of associated genes. With different parameters we obtained different sets of clusters. In particular we focused on the 15 clusters generated with low classification stringency, where one cluster (labeled as 1) grouped together peroxisome and lipid metabolism related categories in addition to other terms. Nevertheless, we did not find the other

associations reported by GENECODIS. These results confirm that while this can be a useful method for an exploratory analysis, it is still distant from providing a full analysis of concurrent functional annotations.
